# Robustness assessment of radiotherapy treatment plans in Switzerland

**DOI:** 10.1016/j.zemedi.2025.03.002

**Published:** 2025-04-21

**Authors:** Hannes A. Loebner, Jenny Bertholet, Paul-Henry Mackeprang, Werner Volken, Michael K. Fix, Peter Manser

**Affiliations:** Division of Medical Radiation Physics and Department of Radiation Oncology, Inselspital, Bern University Hospital, and University of Bern, Bern, Switzerland

**Keywords:** Robustness, Multi-institutional, Audit, VMAT, IMRT, Plan quality, Plan comparison

## Abstract

**Purpose:**

Robustness assessment is an essential part of radiotherapy plan quality assessment. However, it is often not evaluated in photon-based radiotherapy. This study aims to conduct a robustness audit to establish a baseline for the role of plan robustness in Switzerland by assessing and comparing robustness across plans from and clinical workflows in multiple institutions.

**Materials and methods:**

A multi-institutional study involving 11 Swiss institutions was conducted. Each institution provided treatment plans for three cases and completed a questionnaire on treatment planning and assessment of robustness in their clinical practice.

The plans were planned using the Eclipse treatment planning system and utilized intensity-modulated techniques using a 6 MV flattened photon beam for one brain case, and one unilateral and one bilateral head and neck cases, prescribed 60.0 Gy (one phase), 70.0 Gy (two phases) and 70.0 Gy (three phases) to 95% of the target volume, respectively. Institutions used their standard institutional protocols for the provided CT, structures and prescription. Dose distributions were subsequently recalculated in an in-house Monte Carlo (MC) framework incorporating clinically motivated uncertainties associated to patient setup and multi-leaf collimator (MLC) positions. The uncertainties’ impact on the dosimetric plan quality was assessed by evaluating representative target and organ-at-risk (OAR) dose-volume endpoints (e.g. D98% and D2% of the target, mean dose of parallel OARs and near max dose of serial OARs).

**Results:**

Differences in target and OAR dose-volume endpoints in the presence of random patient setup uncertainties (Gaussian distributed with σ = 0.2 cm in the three translational and σ = 0.5° in the three rotational axes) were smaller than ±0.5 Gy. Exceptions were the near max dose-volume endpoints of structures near the target with differences up to ±2.2 Gy for the optic nerve in the brain case. Systematic rotational patient setup uncertainties of ≤3° in either pitch, yaw or roll had similar impact as translational uncertainties ≤0.3 cm in either left-right, superior inferior or anterior-posterior direction with maximal differences in most investigated dose-volume endpoints of 9.0 Gy. Systematic MLC uncertainties of +0.5 mm of all leaves led to an average increase of up to 3.0 Gy in the dose-volume endpoints.

The questionnaire revealed diverse practices in terms of planning and assessment for robustness: all institutions use target and OAR margins, 2/11 use robust optimization and 5/11 regularly perform robustness assessments of treatment plans by recalculating the dose distribution including uncertainties. The importance of robustness in treatment planning was rated ≥8 out of 10 (10 as most important) by 6/11 institutions. The need for better commercial tools to assess or integrate robustness into treatment planning was expressed by 9/11 institutions.

**Conclusion:**

This study presents the first multi-institutional inter-comparison of treatment plan robustness in Switzerland, establishing a robustness baseline for intensity-modulated plans. Despite diverse practices to assess plan robustness and to mitigate the impact of uncertainties on dosimetric plan quality, the robustness to the investigated uncertainties was similar across the plans and cases from all institutes. To foster standardization, we recommend to regularly conduct audits focusing on plan robustness to monitor and reduce inter-institutional variability in handling and assessing plan robustness.

## 1 Introduction

A key step in the radiotherapy treatment workflow is the treatment plan evaluation both in terms of dose metrics and in terms of robustness [1]. While dosimetric plan quality is extensively evaluated in daily clinical workflow, for photon-based treatments this evaluation is usually limited to the nominal scenario where uncertainties are not explicitly assessed. However, during treatment delivery, patient-related uncertainties (e.g., in patient setup [2], [3], [4]) or machine-related uncertainties (e.g., multi leaf-collimator (MLC) positioning uncertainties [5], [6]) influence the delivered dose distribution [5], [7], [8], [9]. In recent years, plan robustness assessment has therefore become a topic of interest [10], [11]. However, in clinical practice, a comprehensive robustness assessment is usually not conducted due to three main reasons. First, robustness assessment imposes an increased workload [9], [12]. Second, there is no standardization regarding which uncertainty types and magnitudes of uncertainties should be assessed, as well as which end-points should be investigated [1], [12]. Third, there is a lack of commercial tools to effectively assess the robustness of a treatment plan [1], [13]. Consequently, there is limited experience in performing comprehensive robustness assessments for photon-based treatments [1]. As a result, there is little knowledge about how plan robustness differs across multiple institutions.

Plan robustness depends on different factors. First, it depends on the treatment technique (e.g. 3D-conformal plans, 3DCRT, or volumetric modulated arc therapy, VMAT) [7], [14]. For instance, a small deviation in the MLC positions minimally affects the clinical target volume dose in 3DCRT plans, whereas it can cause hot and cold dose spots in VMAT plans. Second, plan robustness depends on the planner. Individual variations in the planning approaches between the treatment planners can lead to differences in the resulting treatment plans. Moreover, using different planning protocols, different planning algorithms or different radiation types, will lead to distinct plans, which are expected to have different plan robustness. Even when planning according to the same clinical intent and guidelines [15], [16], [17], usually no two plans for one case are identical and they might not be clinically equivalent. The robustness of these plans is expected to differ as well.

There exist different measures to reduce the occurrence and magnitude of uncertainties in daily clinical practice. For instance, image guided radiotherapy [18], [19], [20] reduces the setup uncertainty, and machine quality assurance (QA) checks reduce the risk of machine miscalibration [21]. Robust optimization directly includes uncertainties at the plan optimization stage [22], with the goal to mitigate their dosimetric impact on the dose distribution. Robust optimization is common in treatment planning of proton-based radiotherapy treatments [23], [24], however it is hardly used for photon-based treatments.

In addition to the above-mentioned measures to reduce and mitigate uncertainties and their impact, external audits also play a crucial role. Audits review different parts of the treatment workflow or treatment machines [25], [26], [27], [28], [29], [30], allowing for comparisons between different institutions to standardize and ensure appropriate treatment quality. They are often required for regulatory purposes [31] and qualification for clinical trials [32], [33]. However, audits regarding plan robustness are scarce. There exists only a recent ESTRO survey which reported on clinical practice for assessing and handling plan complexity and robustness in 126 institutions over 33 countries [13]. However, there is no audit specifically examining the robustness of treatment plans, particularly in Switzerland.

The goal of this study is therefore, to perform a multi-institutional plan robustness inter-comparison in Switzerland. With this audit, we aim to establish a baseline for the role of plan robustness by assessing and comparing the robustness of photon-based radiotherapy treatment plans from multiple institutions for a standardized patient geometry and set of uncertainties that are common in clinical routine. Additionally, we surveyed different institutions about their current practice in terms of treatment planning and robustness assessment. This study complements other multi-institutional studies in Switzerland [28], fostering standardization and raising awareness about the need to consider robustness as an integral part of plan quality.

## 2 Materials and methods

### 2.1 Study design

The study consisted of two parts. In the first part, a multi-institutional robustness assessment of treatment plans to patient setup and machine uncertainties for three clinically motivated cases with treatment sites in the brain and head and neck was performed. The second part consisted of a questionnaire assessing how treatment plan robustness is considered in current clinical practice.

In this study, 29 Institutions across Switzerland were invited to participate by e-mail. To enter the inter-comparison, the following criteria had to be met:

• The institution must perform treatments for at least one of the investigated treatment sites.
• The submitted treatment plans must be provided by a planner with >1 year experience in treatment planning for the investigated treatment sites.
• The treatment planning must be performed in Eclipse (Varian, a Siemens Healthineers Company, Erlangen, Germany) for a TrueBeam treatment machine.

The criteria for treatment planning were required due to the limitations of the employed robustness calculation and evaluation framework [9]. Twenty-one institutions responded and eleven met the inclusion criteria and participated.

### 2.2 Robustness assessment of clinically motivated cases

**Figure 1:**
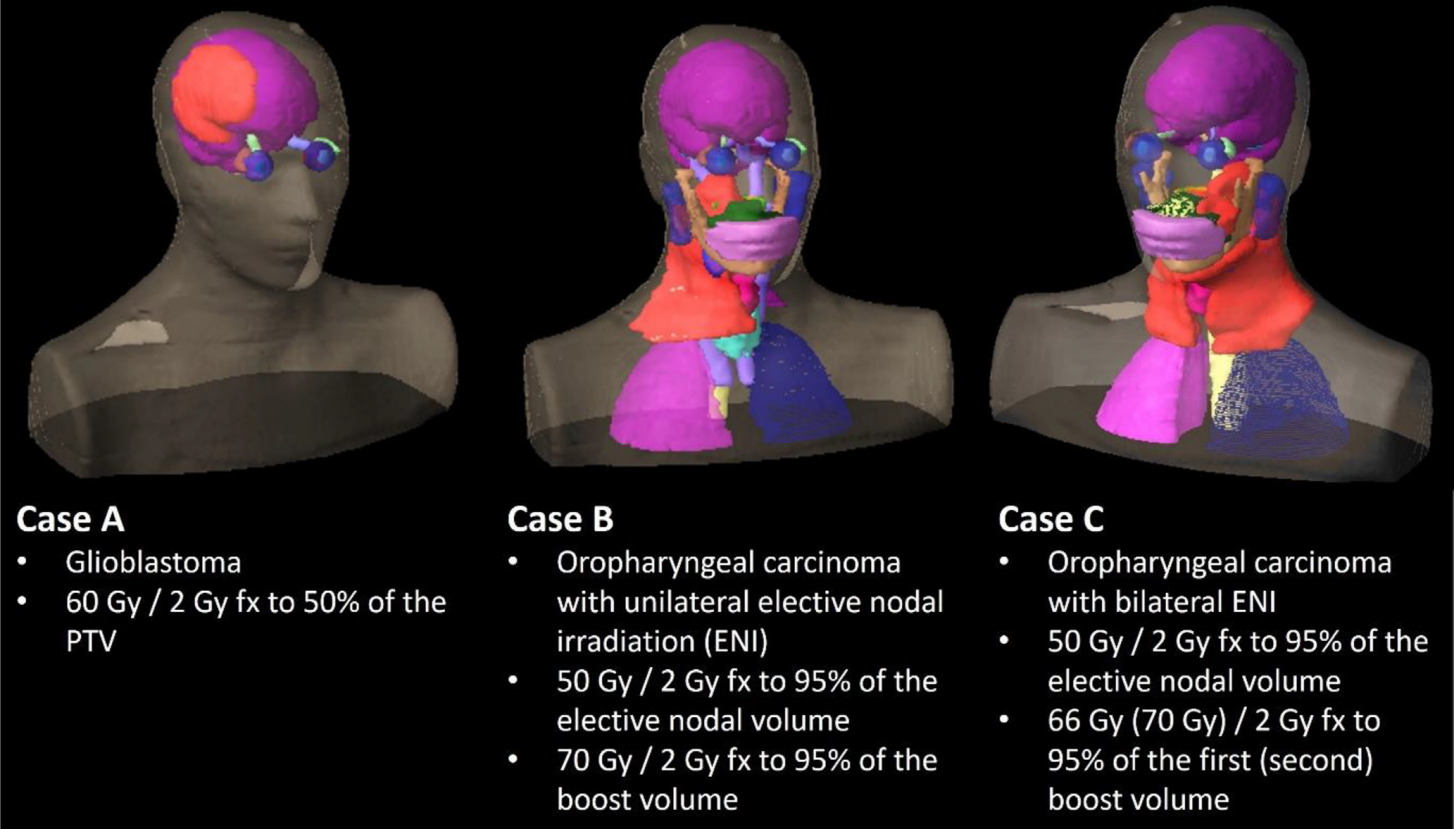
Three clinically motivated cases transferred to the Alderson phantom. The primary PTV is shown in bright red, all other considered OARs are shown in other colors.

The participating institutions were asked to create treatment plans for three clinically motivated cases propagated to an Alderson RT phantom (Rotunda Scientific Technologies LLC, Mansfield, Ohio, USA) [34]. The provided data consisted of a CT, structure set and prescription for each case, illustrated in Fig. 1. Case A was a glioblastoma case with prescription of 60.0 Gy to 50% of the planning target volume (PTV) in 2.0 Gy fractions. Case B and C were oropharyngeal carcinoma with unilateral and bilateral elective nodal irradiation, respectively. The prescriptions were 50.0 Gy to 95% of the elective nodal volume for both cases. Case B had a boost volume prescribed 70.0 Gy to 95% of the volume in 2.0 Gy fractions. Case C had two boost volumes with the first prescribed 66.0 Gy and the second one prescribed 70.0 Gy to 95% of the respective volume in 2.0 Gy fractions. The institutes are asked to normalize their plans to the given respective prescription based on the given PTVs, which were obtained by expanding the clinical target volume (CTV) with a 5.0 mm margin for case A and a 3.0 mm margin for case B and C, trimmed 3.0 mm from the body contour. The plans for the boost volumes were generated separately (no simultaneous integrated boost). Case B and C include planning at risk volumes (PRVs) for the brainstem and spinal cord, which were obtained by a 3.0 mm expansion of the respective structure.

The following treatment techniques were allowed: 3D conformal radiotherapy, intensity modulated radiotherapy (IMRT), VMAT or HyperArc with a 6 MV flattened beam. Deviations from the prescription or adaptations of the structures and margins were not permitted. However, the institutions could introduce help-structures for planning purposes. The treatment plans should conform to their institution-specific target constraints (if not contradicting the prescription) and OAR constraints. The institutions should use the dose calculation algorithm which they use in daily clinical practice. Likewise, the planning technique should be representative of their clinical routine. The participating institutions provided the study organizers the plan-file and the dose distributions of each field/arc in DICOM format.

After collecting the treatment plans, the dose distributions were recalculated using Monte Carlo (MC) within the Swiss Monte Carlo Plan (SMCP) [35], [36] framework. In the calculation, the beam-data of a TrueBeam available at Inselspital was used. The resulting dose distribution represented the MC-calculated nominal scenario (without uncertainties). No renormalization was applied. In the following robustness assessment, the dosimetric impact of the investigated uncertainties on the dose distribution was assessed with respect to the MC-recalculated nominal scenario. This procedure enabled to minimize the impact of the differences in beam-models and dose calculation algorithms between the institutions. In this context, it is worth noting, that TrueBeams have in general very similar dose characteristics [37]. For case B and C always the summed plan (primary + boost or primary + boost 1 + boost 2) was evaluated.

The plans were assessed for robustness to patient setup and MLC position uncertainties described in several uncertainty scenarios (US). To this end, the dose distributions were recalculated including the uncertainties using a previously developed robustness tool [9]. The investigated patient setup uncertainties included both a random and systematic component to mimic intra-fraction motion, inter-fraction patient setup uncertainties and systematic patient setup uncertainties. The random patient setup uncertainties were sampled from a Gaussian distribution with σ = 2.0 mm in anterior-posterior (AP), superior-inferior (SI) and left-right (LR), as well as σ = 0.5° in pitch, yaw and roll [20], [38], [39], [40], [41]. For each plan, one US including only random uncertainties was evaluated. In addition, USs combining random uncertainties as described above with each of the following systematic uncertainties were evaluated: eighteen systematic translational uncertainties in (±2.0 mm, ±3.0 mm, ±5.0 mm) in AP, SI or LR direction and 24 systematic rotational patient setup uncertainties (±0.5°, ±1.0°, ±2.0°, ±3.0°) in pitch, yaw or roll. The uncertainties were based on literature [39], [40], [41], [42], [43], [44], but also included worst case scenarios (e.g., systematic setup uncertainties ±5.0 mm) for explorative purpose.

For MLC uncertainties, only systematic uncertainties were investigated. Previous machine logfile analysis showed negligible random uncertainties without any substantial dosimetric impact even for highly complex techniques such as Dynamic Trajectory Radiotherapy [34], [45], [46]. Systematic uncertainties in MLC position (±0.5 mm, ±1.0 mm, ±2.0 mm) were therefore used to simulate miscalibrations within recommended tolerances [47] and beyond for investigative purposes. A positive uncertainty referred to a wider field opening of the MLC, a negative uncertainty related to a systematic closing of the MLC.

For the nominal scenario and the USs, the calculation voxel size was $0.25*0.25*0.25\;{cm}^{3}$. The statistical uncertainty was <1.1% of voxels which received dose values higher than 50% of the maximum dose following a simulation of approximately 10^8^ primary particles.

The dosimetric impact of the uncertainties was assessed with respect to the nominal scenario by means of dose-volume endpoints for target (D98% and D2% of the CTV) and organs-at-risk (OARs; mean dose, Dmean for parallel OARs, and near max dose, Dcc0.03, for serial OARs).

### 2.3 Questionnaire

The study was accompanied by a 16 items questionnaire, available in the Supplementary material A.1., aiming to assess how robustness is considered in the current clinical practice at each institution. Each institute fully completed the questionnaire, with responses provided by the respective responsible physicist.

## 3 Results

### 3.1 Robustness analysis

The collected plans consisted of (partial arc) VMAT plans, with the exception of 2 institutions submitting HyperArc plans for case A. No IMRT or 3DCRT plan was submitted. In Table 1, a description of the plans is shown. One institution submitted two plans from different planners for each case. Two different types of MLC were used, the HD and Millennium MLC (Varian), differing primarily by the single leaf widths.

**Table 1:** Summary of the collected plans from the eleven institutes.

	**Case A**	**Case B**	**Case C**
**#(partial) arcs: median [range]**	2 [2,3]	2 [2,4] Primary 2 [1,2] Boost	2 [2,5] Primary 2 [2,3] Boost 1 2 [1,2] Boost 2
**Gantry angle range: median [range] in °**	466 [360, 1080]	486 [360, 954] Primary 261 [234, 481] Boost	720 [680, 1800] Primary 627 [360, 720] Boost 1 540 [360, 720] Boost 2
**MU/gantry angle range: median** **[range]**	1.0 [0.5, 2.2]	1.1 [0.5, 1.8] Primary 1.1 [0.7, 1.8] Boost	1.0 [0.4, 1.5] Primary 0.9 [0.7, 1.7] Boost 1 0.9 [0.7, 1.8] Boost 2
**MLC type**	3 HD MLC 8 Millennium MLC	2 HD MLC 9 Millennium MLC	2 HD MLC 9 Millennium MLC

For the MC recalculated nominal scenario, there were substantial differences in the investigated dose-volume endpoints among the different plans (Supplementary Figs. A.2 and A.3). The D98% (D2%) of the PTV varied by up to 6.2 Gy (5.5 Gy) for case A, 13.8 Gy (7.2 Gy) for case B and 11.9 Gy (6.6 Gy) for case C. The large differences in case B and C can be partly attributed to the different dose calculation algorithms used during planning, different beam data between the institutes and the fact that, for some of the plans, the PTV volume was overwritten with water-like HU values during planning. The near-max dose for left optic nerve varied by up to 16.0 Gy for Case A. The right parotid gland mean dose varied by up to 8.6 Gy for Case B and right submandibular gland mean dose varied by up to 23.2 Gy for Case C between the different plans. The right submandibular gland however was partly overlapping with the target; some institute may have decided to prioritize target coverage over OAR sparing.

**Figure 2:**
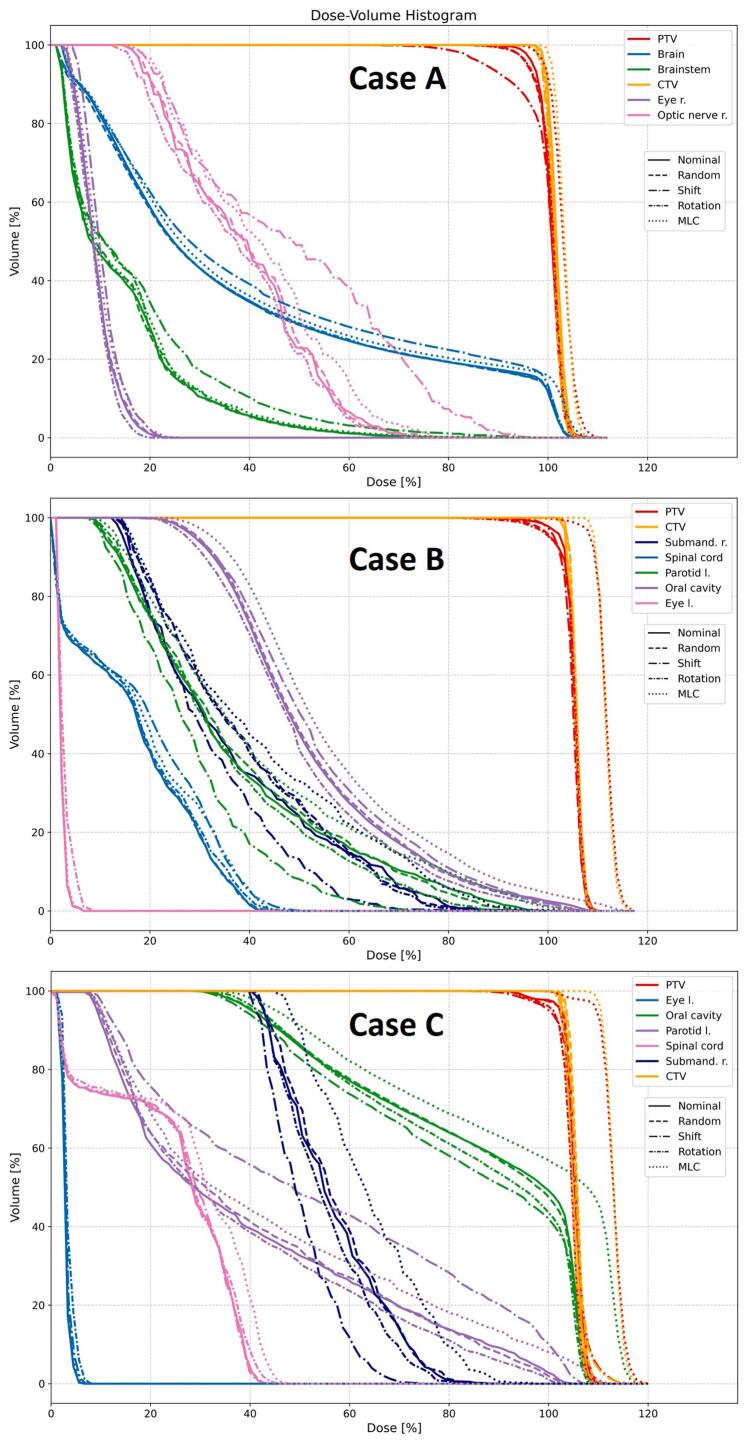
Dose volume histogram of the plans of one representative institute for case A (top), B (middle) and C (bottom) of the nominal and different uncertainty scenarios. “Random” refers to random setup uncertainties only, “Shift” refers to the combination of random setup uncertainties with a systematic lateral shift of 5 mm (to the left of the patient), “Rotation” refers to the combination of random setup uncertainties with a systematic table rotation of 3° (clockwise) and “MLC” refers to a systematic opening of all MLC leaves by 1 mm.

**Figure 3:**
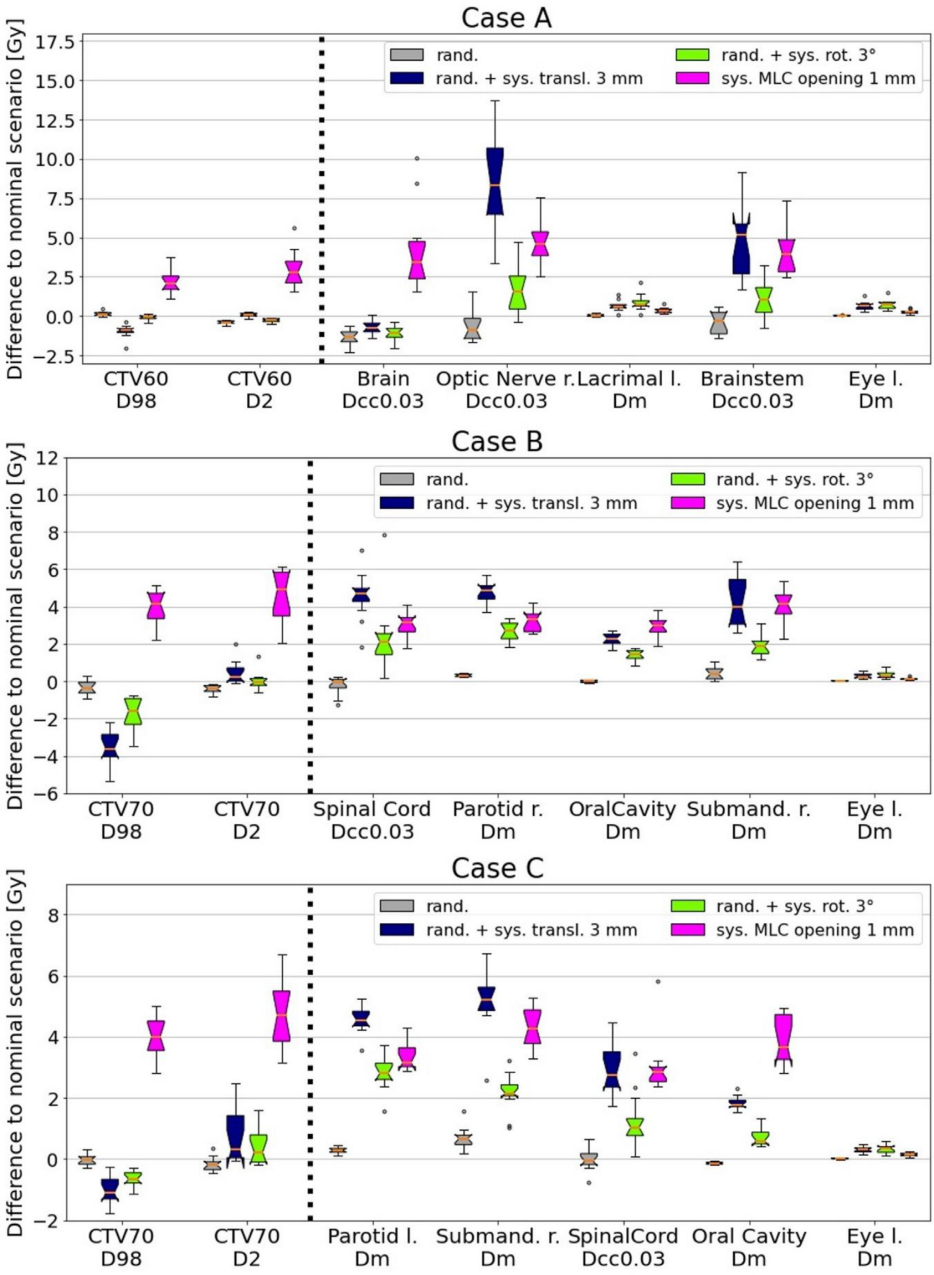
Dose differences with respect to the nominal scenario evaluated for different USs for representative dose-volume endpoints for case A (top), case B (middle) and case C (bottom). Different uncertainty types are visualized in different colors: random patient setup uncertainties only (grey), worst case combination of random + systematic 3 mm translation patient setup uncertainties in AP, SI or LR (blue), worst case combination of random + systematic 3° rotation patient setup uncertainties in pitch, yaw or roll (green), systematic MLC opening of 1 mm (pink). The worst case combination refers to the greatest difference for the respective dose-volume endpoint in the US as compared to the nominal scenario. For instance, for one endpoint this can be the combination of random setup uncertainties with a 3.0 mm systematic setup uncertainty in AP, whereas for another endpoint it could be the combination of random setup uncertainties with a 3.0 mm systematic uncertainty in LR. CTV refers to clinical target volume, submand. stands for submandibular gland and left/right is abbreviated with l./r..

The dose volume histogram of the nominal scenario and different uncertainty scenarios for one representative institute are shown for all cases in Fig. 2. In Fig. 3, the differences with respect to the nominal plans for representative dose-volume endpoints of target and parallel and serial OARs are shown for the different USs and cases. The complete set of results can be found in Supplementary Fig. A.4. The largest differences to the nominal scenario for random patient uncertainties across all plans (indicated in brackets) were observed on near-max dose endpoints, i.e., the right optic nerve (Case A, 1.8 Gy), the brain (Case A, 2.1 Gy), the brainstem (Case A, 1.5 Gy), the spinal cord (Case B, 1.5 Gy), the brainstem (Case B, 1.8 Gy), the brain (Case C, 2.4 Gy) and the brainstem (Case C, 2.2 Gy). For D98% and D2% of the CTV, random patient setup uncertainties caused differences to the nominal scenario within ±0.7 Gy. For the investigated OAR. For the investigated OAR endpoints random patient setup uncertainties caused differences to the nominal scenario within ±0.5 Gy for all cases and plans.

The impact of the worst case combination of random with systematic patient setup uncertainties of 3.0 mm (any direction) or with 3.0° (any axis) on the investigated dose-volume endpoints compared to the nominal scenario remained below 9.0 Gy for most investigated endpoints, cases and plans. The only exceptions were the near max dose for the right optic nerve and the brainstem of case A: these structure are in close proximity to the target and deviated by up to 14.1 Gy and 9.2 Gy compared to the nominal scenario. The worst case combination refers to the greatest difference for the respective dose-volume endpoint in the US as compared to the nominal scenario. In more detail, structures near the target experienced greater variations in their dosimetric properties with respect to the nominal scenario than structures further away from the target and beam path. For instance, mean dose to the eye for case B and C had differences by <1.0 Gy, whereas the mean dose for the right parotid (case B) and the right submandibular gland (case C) had maximum differences of 7.0 Gy with respect to the nominal scenario. A visual representation of the impact of random combined with systematic setup uncertainties can be found in the Supplementary material (Supplementary Fig. A.5).

For structures close to the target and beam path, systematic MLC miscalibration of 0.5 mm opening lead to an average increase of 3.0 Gy across the investigated dose-volume endpoints, for all cases and plans. For structures further away from the target and beam path (e.g., the eyes in case B and C), they remained <1.0 Gy. A systematic miscalibration of 1.0 mm MLC opening lead to increases of up to 10.0 Gy.

With respect to the used treatment technique (2 HyperArc plans and 9 VMAT plans for case A), no clear treatment technique dependence of plan robustness was observed (see Supplementary Fig. A.4).

### 3.2 Questionnaire

**Figure 4:**
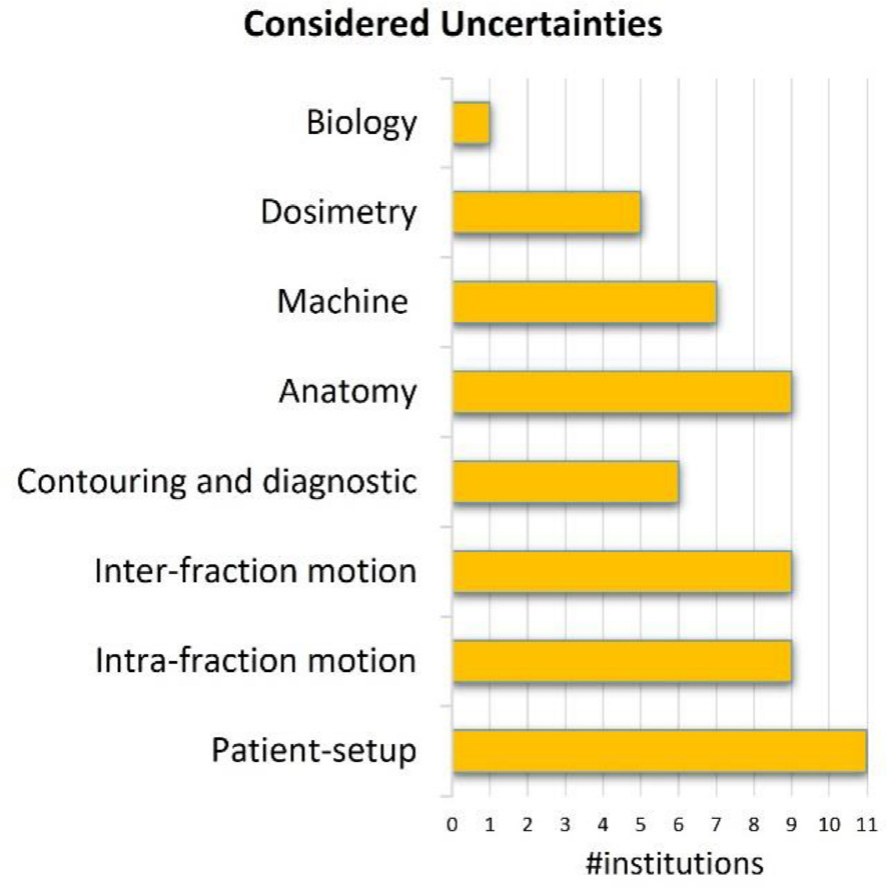
Overview of different areas of uncertainty and how many institutions consider them directly or indirectly (Q2).

When asked to rate the importance of treatment plan robustness to patient setup and machine uncertainties in their treatment planning process (Q1), 6/11 responders gave a score ≥ 8/10 (range 1–10, with 10 as “high priority”, 5 as “indirectly considered”, and 1 as “low priority”); the remaining responders scored it 5/10. Fig. 4 shows an overview of uncertainty areas and by how many institutes they are considered (Q2). Patient setup uncertainties were considered by all institutions, whereas biological uncertainties (e.g., uncertainty in the tumor response due to hypoxia) was only considered by one.

All institutions used CTV-PTV margins, which were derived from literature (Q3). Five institutions further indicated considering clinically-observed patient setup uncertainties in their margin recipes, and three indicated using formula-based calculations in their margin calculation [48]. However, 6/11 institutions allowed changes of these margins for plan optimization purposes (Q4). Regarding OARs (Q5), all except one institution used planning at risk volumes (PRVs), which were either based on literature (6/11) or derived from clinically-observed patient setup uncertainties (5/11) while one institution used a formula-based approach for PRVs (Q6).

**Figure 5:**
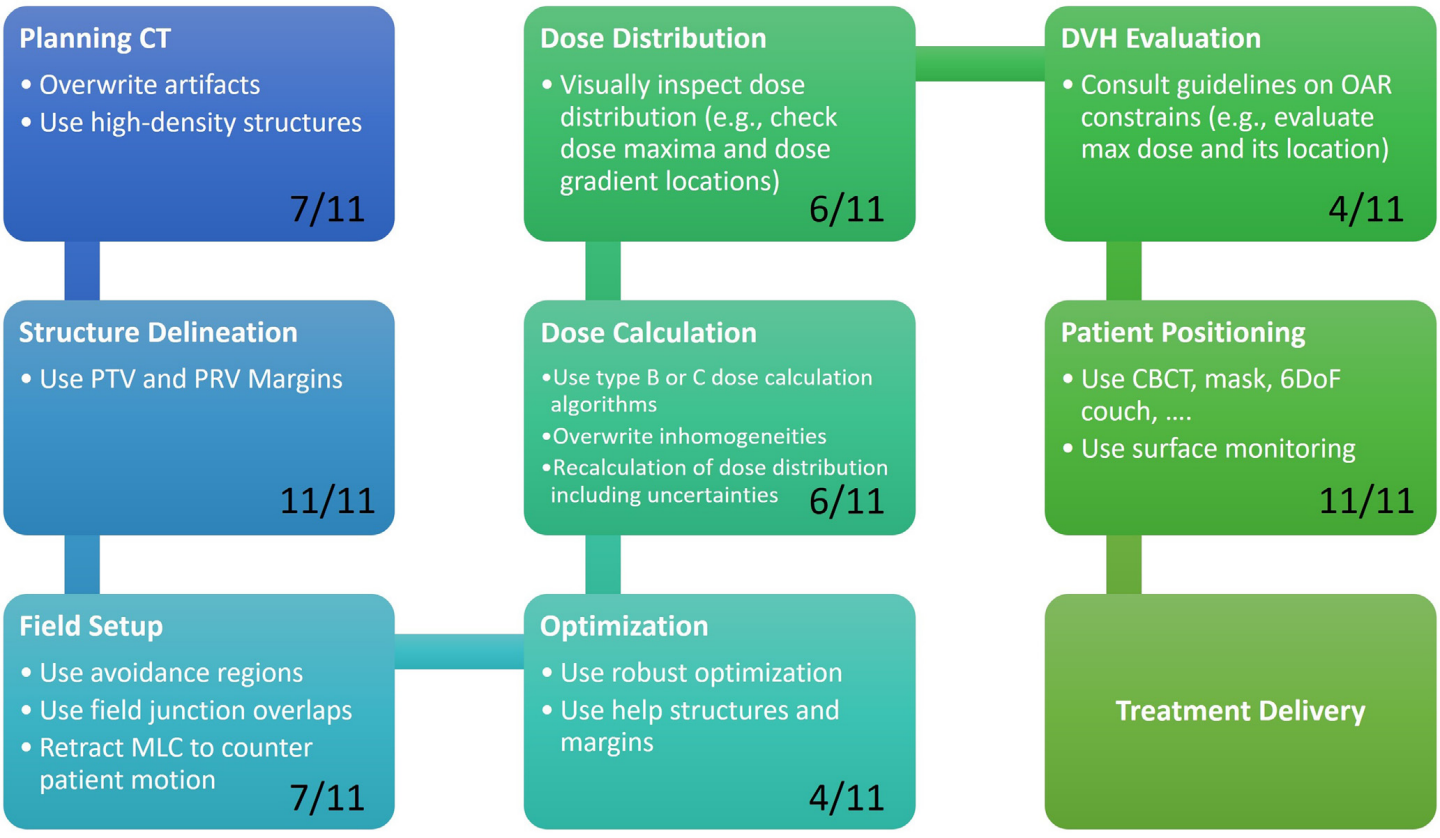
Treatment planning process including potential ways to mitigate uncertainties and/or improve the robustness. The black number indicates how many institutions indicated taking action at these respective steps (Q10).

In Fig. 5, a schematic overview of a typical treatment planning process is visualized including different possibilities to consider robustness at the different stages (Q10). The black number indicates how many institutions took measures to reduce or mitigate uncertainties and/or improve robustness at the respective stage. Of note, two institutions explicitly performed robust optimization and five perform robustness assessment of a treatment plan by recalculating the dose distribution including uncertainties (Q8 and Q10).

Among the different professions involved in the treatment planning process, it was physicists (11/11) and radiation therapists (RTTs) (7/11) who usually raised concerns regarding robustness to patient setup or machine uncertainties (Q11). This was followed by planner/dosimetrists (6/11) and the prescribing physician (3/11). At none of the institutions, the tumor board raised concerns related to plan robustness, however for approximately half of the institutions (5/11) the personnel involved in plan review (usually physicians, e.g. in a morning rapport setting), raised concerns related to plan robustness.

Robustness assessment can be based on a variety of factors for the different institutions (Q8): three institutions evaluated the distance between target and OARs to assess if additional measures to mitigate the impact of uncertainties on the dose distributions need to be taken. Five institutions included additionally dosimetric information into account: they used geometrical considerations based on contours and planned dose distribution (e.g., the distance of OARs to high dose regions or dose gradients) as a basis for a robustness assessment. Four institutions recalculated the dose distribution, including uncertainties, to study their impact in a robustness assessment. Four institutions reported that they do not actively assess the robustness of treatment plans to patient setup and machine uncertainties. Robustness assessments in the clinic have led to replanning or plan adaptations for all but two institutions (Q12). Notably, when a robustness assessment was performed, one institution reported that every second plan was subject to replanning or adaptation due to robustness concerns (Q13). Possible actions following a robustness assessment included (Q12): raising awareness by alerting the relevant personnel and book-keeping (8/11), additional imaging (e.g., CBCT for patient setup 9/11), additional or different patient immobilization (6/11) and replanning with (3/11) and without (9/11) robust optimization.

On the immediate outcome of this study, 9/11 wished for better commercial tools to consider robustness in the treatment planning process by means of facilitated plan robustness evaluation or robust optimization (Q15). Three institutions stated that this study changed their view on robustness (Q14). On the question if their consideration of robustness during treatment planning will increase based on this study (Q16), three responded with a score of ≥ 5, while the rest answered with a score ≤3 (range 1–10, with 1 as “treatment planning process will stay as is”, 5 as “I am more aware now” and 10 as “I want to extend the current treatment planning process to actively consider robustness”).

## 4 Discussion

This study reports on the first multi-institutional effort to assess and compare the robustness of radiotherapy treatment plans across institutions in Switzerland and to evaluate how robustness is considered in daily clinical practice.

The submitted treatment plans evaluated in the robustness assessment included different techniques and field setups, which led to substantial differences in plan quality in the nominal scenario (e.g., right submandibular gland mean dose of case C varied by 23.2 Gy across the plans). Prioritization of OAR sparing was likely different among planners. However, despite these differences, the robustness of the plans was similar: random patient setup uncertainties across the investigated endpoints were generally below ±0.7 Gy for all cases and plans, independent of the treatment technique.

Regarding the impact of the investigated uncertainties, systematic rotational patient setup uncertainties up to 3.0° had a similar impact on the dose volume parameters as systematic translational patient setup uncertainties up to 3.0 mm in SI, AP or LR direction. The impact of these uncertainties in the investigated endpoints remained below 9.0 Gy. For machine uncertainties, a MLC miscalibration of 0.5 mm lead to an increase/decrease of approximately 3.0 Gy across most investigated endpoints, whereas a miscalibration of 1.0 mm lead to an increase/decrease of up to 10.0 Gy. Exceptions were found for the structures further away from the primary beam path (e.g., the eyes for case B and C), where the increase in mean dose was <0.5 Gy. It is important to note, that these structures received <0.5 Gy for the investigated dose-volume endpoints in the nominal scenario. In general, dose-volume endpoints of structures in near proximity to the target or close to the primary beam path (e.g., submandibular glands), as well as near max dose endpoints (near max dose for brainstem) were most affect by the uncertainties, as compared to structures further away (e.g., the eyes). This is to be expected since this is where large gradients in the dose distribution occur. These findings are also in line with previous robustness assessments for VMAT plans: a similar impact of patient-setup uncertainties on the investigated endpoints was reported, as well as that already a small miscalibration of the MLC can lead to substantial differences in the dose distribution [5], [9], [11].

The selected USs included both random and systematic patient setup uncertainties observed in clinical practice, along with worst-case scenarios [18], [19], [20], [38], [42], [43], [44]. It is important to note that in clinical practice, the magnitude of patient setup uncertainties is closely linked to the applied setup technique. CBCT and mask fixation for head and neck cancer patients [18], [38], [43], combined with a 6-degrees of freedom couch can substantially reduce patient setup uncertainties and improve setup reproducibility [44]. For the selection of the MLC position uncertainties, established tolerance thresholds [47], [49], [50], [51], [52] were chosen to assess the impact of realistic miscalibrations [53]. However, it has to be mentioned, that the daily variation in MLC accuracy has been found to be within ±0.1 mm [54], but the greatest source of error are miscallibrations [53]. Additionally, we explored USs beyond these limits (>1 mm) for investigative purposes, though they should be considered extreme scenarios [5], [49], [50], [55].

The survey revealed substantial differences in how institutions address treatment plan robustness in radiotherapy. All institutions considered patient setup uncertainties, but only one responded to account for uncertainties in tumor response due to biological uncertainties such as hypoxia. Common practice included using CTV-PTV margins derived from literature, clinically-observed patient setup uncertainties, and formula-based calculations. Most institutions employed PRVs. From the professions involved in the treatment planning process, it was mostly Physicists and RTTs who raise concerns regarding robustness to patient setup and machine position uncertainties, while plan robustness was discussed at plan review by half of the institutions. It should be noted that the questionnaire was filled-out by physicists which might have caused a bias in this particular response. When a robustness assessment is conducted, the institutions indicated that potential actions could involve additional setup imaging and patient fixation devices to reduce patient setup uncertainties or replanning to improve plan robustness. It is important to note, that these additional actions however, prolong the treatment workflow, can increase the dose to the patient (by additional imaging) [56] and may cause patient discomfort [57], [58]. A wish for better commercial tools to facilitate robustness evaluation and optimization was expressed by a majority of institutions, which coincides with the outcome of previous studies [1], [13].

There are some limitations to this study. First, the inclusion criteria restricted the number of participating institutions. Future investigation should extend to different treatment machines and treatment planning systems to enable more institutions to participate. Second, the dose distributions (with and without uncertainties) were recalculated with beam-data and a dose calculation algorithm not specific to the respective institution. In this context, it is however worth noting, that TrueBeams have in general very similar dose characteristics [37]. The institution-specific dose calculation algorithms were not always available and the beam data was often treated confidentially. However, the robustness assessment was performed by evaluating the impact of the uncertainties as differences with respect to the nominal scenario so that institution-specific beam data settings, such as a dosimetric leaf-gap, and dose calculation algorithm specific features, are expected to cancel out in the comparison to the recalculated nominal scenario. Moreover, by using MC as the dose calculation algorithm, we included the gold-standard of radiotherapy dose calculation [59]. Third, the presented robustness assessment focused on only two treatment sites, which could be seen as a limitation. Nonetheless, these treatment sites are of particular interest, as they are considered challenging due to the numerous OARs in close proximity to the target and planners must manage conflicting goals (e.g., when target and OAR overlap) and need to balance dosimetric plan quality with dosimetric robustness. However, our robustness assessment could be straightforwardly extended: by transferring additional tumor entities to the open-source available Alderson RT phantom library [34], different treatment sites could be added to this robustness study and investigated in future studies. Using this approach of transferring clinically-observed cases to the Alderson RT phantom further has the advantage to facilitate a comparison with multiple institutions as no ethics approval is needed. Moreover, measurements to verify the deliverability of the created plans could be added easily. Lastly, the questionnaire might be subject to responder bias. As it was filled out only by medical physicists, their answers might not accurately represent the views of all professions. For example, physicians might have provided different responses for the question regarding awareness of plan robustness (Q11).

The present study inscribes itself in an established tradition for multi-institutional studies. In Switzerland, clinical [60] and technical [61] audits (required by law) are conducted on a regular basis. Moreover, voluntary audits such as the Swiss Society of Radiobiology and Medical Physics (SSRMP)-initiated thermoluminescent dosimeter (TLD) intercomparison [28] are carried out annually with institutions taking part with the goal to improve quality and standardization across Switzerland. The present study complements these existing audits by presenting the first audit of plan robustness in Switzerland. In a wider frame, the results of the present study agree with the findings of the international 2020 ESTRO survey on plan complexity and robustness [13]: the use of PTV and PRV margins (98% of participating institutions), as well as the use of robust optimization (21% of participating institutions) and robustness assessments (13% of participating institutions) were on similar levels as in our study.

The present study can serve as a baseline for future plan robustness audits. Repetition of this robustness audit can reveal if there are improvements in plan robustness or a reduction in variation between the institutions, i.e. better inter-institutional consistency regarding plan robustness. Especially when introducing new treatment techniques, a comparison to this study, serving as baseline, could reveal if the dosimetric benefits of the new technique over the standard of care persists in the presence of uncertainties [11]. Furthermore, the presented study holds the potential to reinvestigate current tolerance levels on the patient and on the machine side [47] for currently used treatment techniques.

Given the results of this study, particularly the responses to question 14–16, suggests repeating this robustness intercomparison regularly, and to also extend it to different planning systems, treatment machines and treatment sites with the goal to monitor and standardize handling and assessing plan robustness.

## 5 Conclusion

This study presented the first multi-institutional robustness inter-comparison in Switzerland for photon-based radiotherapy. While dosimetric plan quality of the investigated plans showed substantial variability in the investigated dose-volume endpoints, and robustness during treatment planning is considered differently at different institutions, the dosimetric robustness to the considered uncertainties was similar for all plans of the three investigated cases. This study can serve as a baseline for future plan robustness investigations. Based on the results of this study, we recommend to regularly conduct such robustness audits to monitor and reduce inter-institutional variability.

## Data Availability Statement

Research data are not available at this time.

## CRediT authorship contribution statement

**Hannes A. Loebner:** Writing – review & editing, Writing – original draft, Visualization, Software, Project administration, Methodology, Investigation, Formal analysis, Conceptualization. **Jenny Bertholet:** Writing – review & editing, Supervision, Methodology, Investigation, Conceptualization. **Paul-Henry Mackeprang:** Writing – review & editing, Methodology, Conceptualization. **Werner Volken:** Writing – review & editing, Methodology. **Michael K. Fix:** Writing – review & editing, Supervision, Resources, Project administration, Methodology, Conceptualization. **Peter Manser:** Writing – review & editing, Supervision, Resources, Project administration, Methodology, Conceptualization.

## Declaration of competing interest

The authors declare the following financial interests/personal relationships which may be considered as potential competing interests: This work was supported by Varian, a Siemens Healthineers Company and the SSRMP Research Grant 2023.
