# Supplementary material for: Robustness assessment of radiotherapy treatment plans in Switzerland
Source: Z Med Phys. 2025 Apr 21;36(1):47–59. doi: 10.1016/j.zemedi.2025.03.002 (PMC12901509; doi:10.1016/j.zemedi.2025.03.002)
Supplement: Supplementary Data 1 [file mmc1.pdf]

Supplementary Material for

Robustness Assessment of Radiotherapy Treatment Plans in Switzerland

Hannes A. Loebner[1], PhD, Jenny Bertholet[1], PhD, Paul-Henry Mackeprang[1], MD, PhD, Werner Volken[1], PhD, PhD, Michael K. Fix[1], PhD and Peter Manser[1], PhD

[1]Division of Medical Radiation Physics and Department of Radiation Oncology, Inselspital, Bern University Hospital, and University of Bern, Bern, Switzerland.

Supplementary material A.1: Questionnaire

The questionnaire aims to determine how treatment plan robustness is considered at your center in current clinical practice. To answer the questions please include all relevant professions involved in the treatment planning process.

GENERAL

1. **How important is treatment plan robustness to patient setup and machine uncertainties in your treatment planning process?**
(Please mark the corresponding cell with an x)

1<br>low<br>priority	2	3	4	5<br>indirectly<br>considered	6	7	8	9	10<br>high<br>priority

2. **What uncertainties are usually considered at your institution?**
(Please indicate one or more applicable options)
   - o Patient setup uncertainties
   - o Intra-fraction patient motion uncertainties
   - o Inter-fraction patient motion uncertainties
   - o Contouring and diagnostic uncertainties
   - o Uncertainties related to patient anatomy changes (e.g., tumor shrinkage)
   - o Treatment machine uncertainties
   - o Dosimetric uncertainties (e.g., output, range uncertainty)
   - o Biological uncertainties (e.g., uncertainties in tumor response due to hypoxia)
   - o Other: ___________________________________

3. **How are the clinical target volume (CTV) - planning target volumes (PTV) margins determined in your institution?**
(Please indicate one or more applicable options)
   - o We do not use a PTV
   - o Literature based
   - o Translating setup uncertainties into margins

- o Formula based (e.g., van Herk or others)
- o Other: ____________________________________

4. Do you allow changes of the CTV-PTV margins for optimization purposes?

- o Yes
- o No

5. Do you use planning organ-at-risk volumes (PRVs)?

- o Yes
- o No

6. How are the PRV margins determined in your institution?

(Please indicate one or more applicable options)

- o We do not use PRVs
- o Literature based
- o Translating setup uncertainties into margins
- o Formula based (e.g., van Herk or others)
- o Other: ____________________________________

PATIENT SETUP AND MACHINE UNCERTAINTIES

7. Do you consider robustness to patient setup and to machine uncertainties in the treatment planning process and, if yes, to which extent?

(Please indicate one or more applicable options)

- o Not considered
- o Use of structure margins determined from in-house cohort analysis
- o Adapt field and collimator setup (e.g., field junction overlaps, retracted MLC for tangential fields in 3D-CRT planning)
- o Perform visual robustness evaluation (e.g., visual inspection of dose distribution)
- o Perform calculation of dose distributions for different uncertainty scenarios (patient setup and machine uncertainties)
- o Use robust optimization
- o Other: ____________________________________

8. What is the basis for your robustness assessment (regarding patient setup and machine uncertainties)?

(Please indicate one or more applicable options)

- o The robustness assessment is based on geometrical considerations based on the contours: e.g., distance between target and OARs
- o The robustness assessment is based on geometrical considerations based on contours and planned dose distributions
- o The robustness assessment is based on recalculated dose distributions with incorporation of the uncertainty
- o The robustness to patient setup and machine uncertainties is not actively assessed
- o Other: ____________________________________

9. **If you perform a robustness assessment, which uncertainties are you currently considering?**
   (Please indicate and/or fill out the blank spaces.)
   - We do not perform a robustness assessment.
   - Patient setup left right
     - Not considered
     - Up to ______mm
   - Patient setup anterior posterior
     - Not considered
     - Up to ______mm
   - Patient setup cranial caudal
     - Not considered
     - Up to ______mm
   - Pitch
     - Not considered
     - Up to ______°
   - Roll
     - Not considered
     - Up to ______°
   - Yaw
     - Not considered
     - Up to ______°
   - Gantry rotation
     - Not considered
     - Up to ______°
   - Table rotation
     - Not considered
     - Up to ______°
   - Collimator rotation
     - Not considered
     - Up to ______°
   - Multi-leaf collimator leaf position
     - Not considered
     - Up to ______mm
   - Jaw position
     - Not considered
     - Up to ______mm
   - Output
     - Not considered
     - Up to ______%
   - Other
     - Up to ______[____]

Robustness Assessment in the Treatment Planning Process

In case you consider plan robustness during treatment planning:

10. **At what stage in the treatment planning process is robustness considered?**
    (Please indicate one or more applicable options)

- Planning CT (e.g., overwrite artifacts, use high density structures)
- Contouring / Structure Delineation (e.g., use margins)
- Employment of Help-Structures/Avoidance-Regions
- Field setup (e.g., use avoidance regions, retract MLC for tangential fields in 3D-CRT planning, use field junctions overlap)
- Optimization (e.g., use robust optimization)
- Dose calculation
- Visual inspection of the dose distribution (e.g., inspect Dmax and dose gradients)
- Dose-Volume-Histogram parameter inspection (e.g., Dmax of certain organs)
- Patient positioning (e.g., use of CBCT, mask, 6DoF couch, surface monitoring)
- Other: ___________________________________

11. In your treatment planning process, who is usually raising concerns regarding or considering robustness to patient setup and machine uncertainties?
(Please indicate one or more applicable options)

- RTTs
- Planners/Dosimetrists
- Physicists
- Prescribing Physicians
- Tumor Board
- Plan Review (usually physicians, e.g. in a morning rapport setting)
- Other: ___________________________________

12. What are possible actions following a robustness assessment (even if it is not labelled "robustness") in your treatment planning process?
(Please indicate one or more applicable options)

- Raising awareness/alert (book keeping, information of relevant personnel)
- Additional imaging, e.g. CBCT for setup
- Additional or different patient immobilization, e.g. 5-point masks
- Re-planning (without robust optimization)
- Re-planning with robust optimization
- Other: ___________________________________

13. How often does a robustness assessment lead to re-planning or adaptations (e.g., in field setup, or optimization) at your institution?
(Please mark the corresponding cell with an x)

1 never	2	3	4	5 every second time	6	7	8	9	10 every time

Immediate impact of this study

14. Have your views on plan robustness changed after participating in this questionnaire?

- No
- Yes

15. **Do you wish for better commercial tools to include robustness considerations in your treatment planning process?**
    - No
    - Yes

16. **Will your consideration of robustness during treatment planning increase?**
    (Please mark the corresponding cell with an x)

1 treatment planning process will stay as is	2	3	4	5 I am more aware now	6	7	8	9	10 I want to extend the current treatment planning process to actively consider robustness

Supplementary figure A.2:

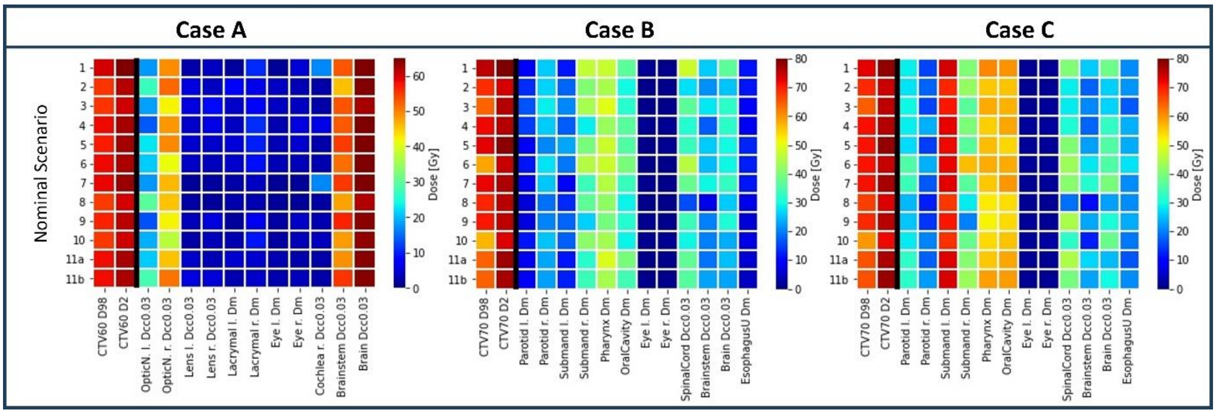

Figure A.2: Dose-volume endpoints for the MC recalculated dose distribution of the submitted treatment plans for case A (left), case B (middle) and case C (right) in the nominal scenario. One institution submitted two plans, denoted with 11a and 11b. For case B and C the dose-volume endpoints of the summed plan (primary + boost) are visualized. Target dose-volume endpoints are separated from OAR dose-volume endpoints by a black vertical line.

Supplementary figure A.3:

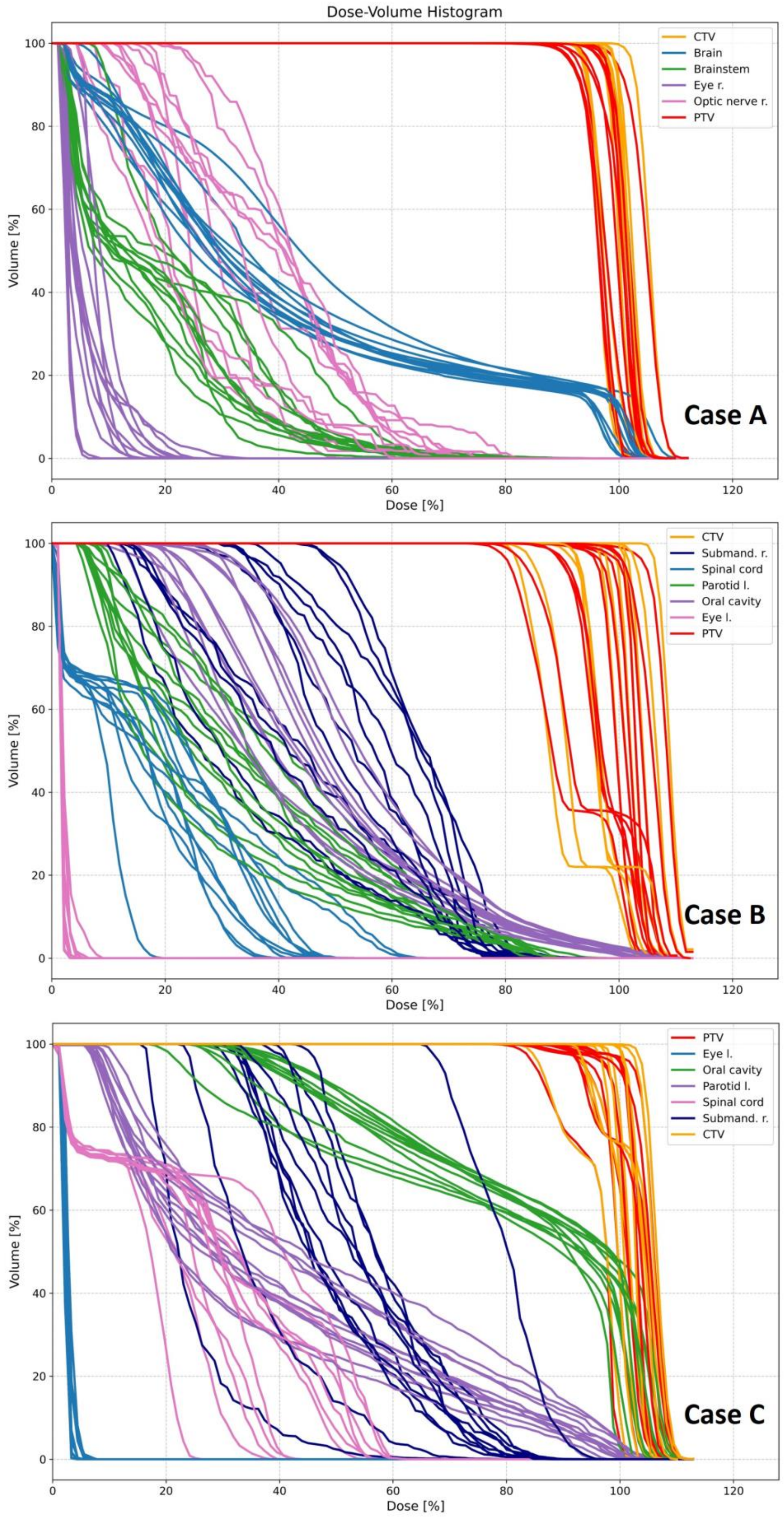

Figure A.3: Dose-volume histograms for the MC recalculated dose distribution of the submitted treatment plans for case A (top), case B (middle) and case C (bottom) in the nominal scenario. For case B and C the dose-volume histograms of the summed plan (primary + boost) are visualized. The dose is given in percent of the prescribed dose. For case B and C, the PTV volume was overwritten with water-like HU values during planning for some institutes leading to a "step" in the target volume DVH. The institutes followed their institutional planning guidelines, including different prioritization of dose volume endpoints, which can lead to substantial differences (e.g., right submandibular gland case C).

Supplementary figure A.4:

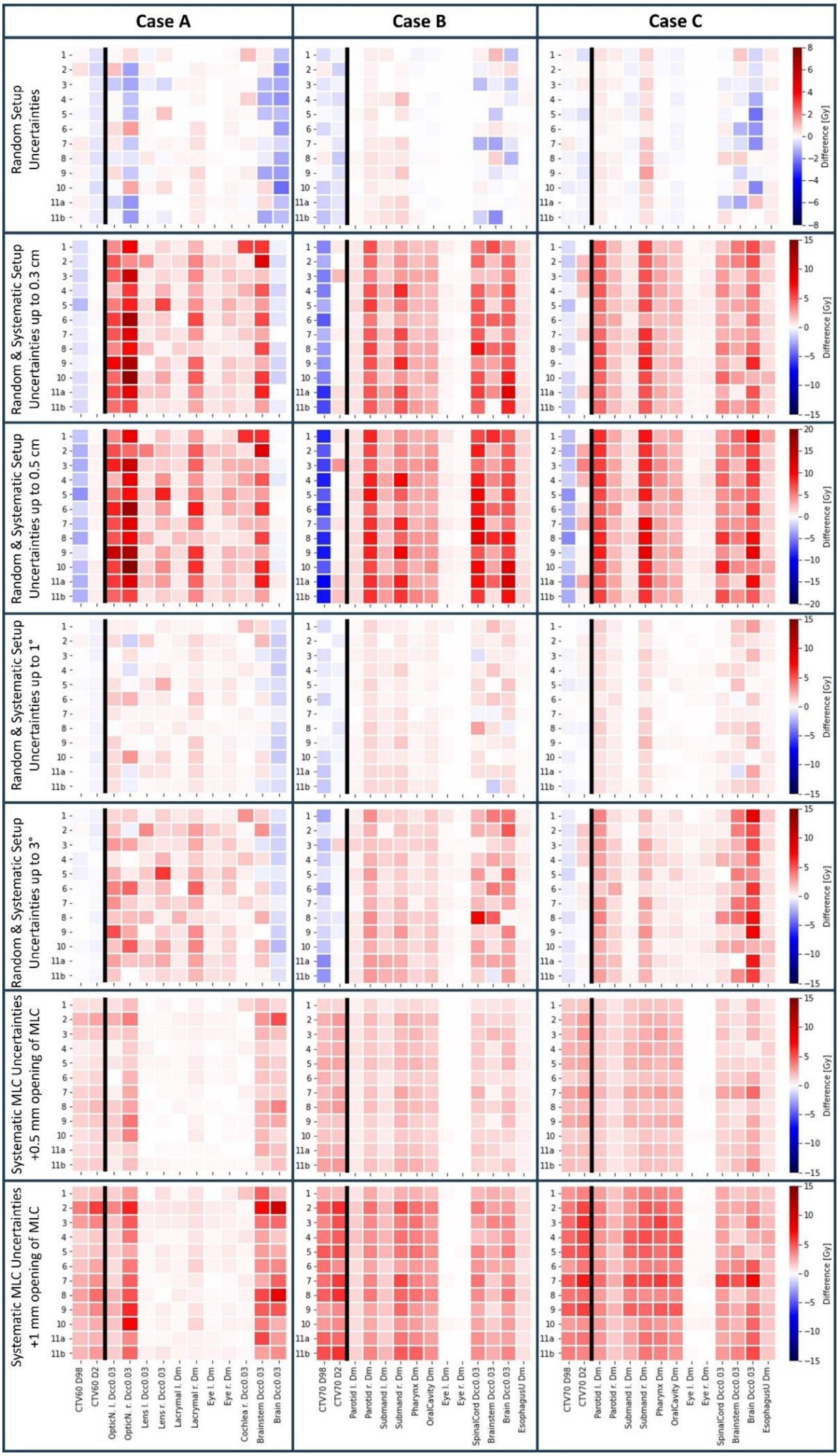

Figure A.4: Impact of the different uncertainties in dose-volume endpoints for the submitted treatment plans visualized as the respective difference between the nominal and USs for case A (left), case B (middle) and case C (right). One institution submitted two plans, denoted with 11a and 11b. For case B and C the differences in dose-volume endpoints of the summed plan (primary + boost) and the summed plan of the nominal scenario are visualized. Target dose-volume endpoints are separated from OAR dose-volume endpoints by a black vertical line. The differences in endpoints for the combination of random + systematic patient setup uncertainties referrers to the respective worst-case combination. The worst-case combination refers to the greatest difference for the respective dose-volume endpoint in the uncertainty scenario as compared to the nominal scenario. For instance, for one endpoint this can be the combination of random setup uncertainties with a 3.0 mm systematic setup uncertainty in AP, whereas for another endpoint it could be the combination of random setup uncertainties with a 3.0 mm systematic uncertainty in LR. CTV refers to clinical target volume, submand. stands for submandibular gland and left is abbreviated with l.

Supplementary figure A.5:

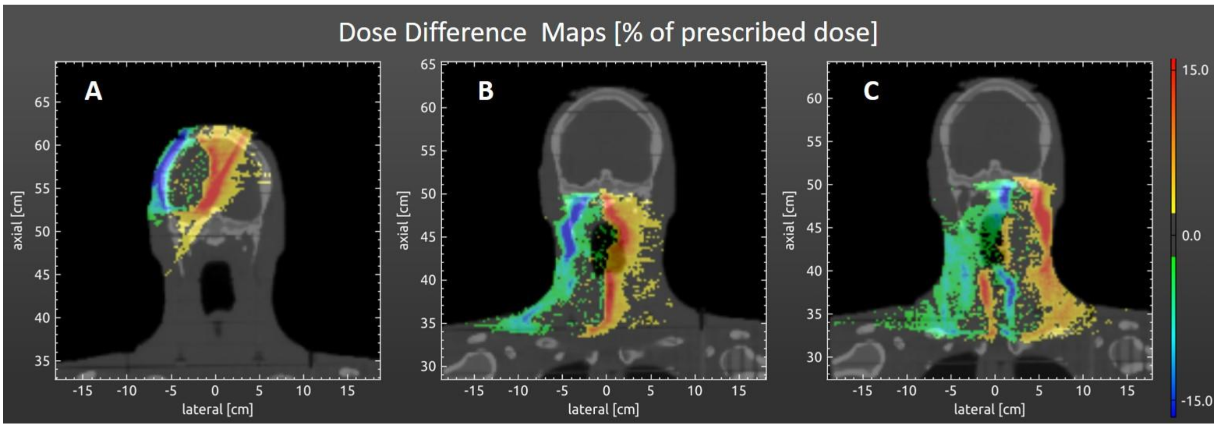

Figure A.5: Dose difference maps visualizing the impact of random combined with systematic lateral shift (towards the left of the patient) uncertainty of 3 mm in percent of prescribed dose for case A (left), B (middle) and C (right). For case B and C the impact on the summed plan (primary + boost) is visualized.
